# Investigating the role of FADS family members in breast cancer based on bioinformatic analysis and experimental validation

**DOI:** 10.3389/fimmu.2023.1074242

**Published:** 2023-04-12

**Authors:** Tingting Zhao, Pingping Gao, Yanling Li, Hao Tian, Dandan Ma, Na Sun, Ceshi Chen, Yi Zhang, Xiaowei Qi

**Affiliations:** ^1^ Department of Breast and Thyroid Surgery, Southwest Hospital, Third Military Medical University, Chongqing, China; ^2^ Academy of Biomedical Engineering, Kunming Medical University, Kunming, China; ^3^ Yunnan Cancer Hospital & The Third Affiliated Hospital of Kunming Medical University & Yunnan Cancer Center, Kunming, China

**Keywords:** breast cancer, fatty acid desaturase, bioinformatics, prognosis, immunity

## Abstract

Breast cancer (BC) is the most common malignant tumor in women worldwide. Emerging evidence indicates the significance of fatty acid metabolism in BC. Fatty acid desaturase (FADS) is closely associated with cancer occurrence and development. Here, bioinformatic analysis and experimental validation were applied to investigate the potential functions of FADS in BC. Several public databases, including TCGA, GEO, HPA, Kaplan–Meier plotter, STRING, DAVID, cBioPortal, TIMER, TRRUST, and LinkedOmics were used to determine mRNA/protein expression levels, prognostic significance, functional enrichment, genetic alterations, association with tumor-infiltrating immune cells, and related transcription factors and kinases. BC tissues showed higher and lower mRNA expression of FADS2/6/8 and FADS3/4/5, respectively. FADS1/2/6 and FADS3/4/5 showed higher and lower protein expression levels, respectively, in BC tissues. Moreover, FADS1/7 up- and FADS3/8 down-regulation predicted poor overall and recurrence-free survival, while FADS2/5 up- and FADS4 down-regulation were associated with poor recurrence-free survival. Receiver operating characteristic curves revealed that FADS2/3/4/8 were indicative diagnostic markers. FADS family members showing differential expression levels were associated with various clinical subtypes, clinical stages, lymph node metastasis status, copy number variants, DNA methylation, and miRNA regulation in BC. The mRNA expression level of FADS1/2/3/4/5/7/8 was observed to be significantly negatively correlated with DNA methylation. FADS1/2 upregulation was significantly correlated with clinical stages. FADS1/4 expression was obviously lower in BC patients with higher lymph node metastasis than lower lymph node metastasis, while FADS7/8 expression was obviously higher in BC patients with higher lymph node metastasis than lower lymph node metastasis. FADS family members showed varying degrees of genetic alterations, and Gene Ontology and KEGG pathway enrichment analyses suggested their involvement in lipid metabolism. Their expression level was correlated with immune cell infiltration levels. FADS2 was chosen for further validation analyses. We found FADS2 to be significantly over-expressed in clinical BC tissue samples. The proliferation, migration, and invasion abilities of MDA-MB-231 and BT474 cells were significantly reduced after FADS2 knockdown. Furthermore, FADS2 may promote the occurrence and development of BC cells *via* regulating the epithelial–mesenchymal transition (EMT) pathway. Altogether, our results suggest that FADS1/2/3/4 can serve as potential therapeutic targets, prognostic indicators, and diagnostic markers in patients with BC.

## Introduction

Cancer is the most serious public health concern across the globe. According to the data reported by Global Cancer Statistics 2020, in women, breast cancer (BC) has surpassed lung cancer as the most commonly diagnosed cancer (11.7% of total cases) (1). One of the characteristics of cancer cells is that they rebuild their metabolism to produce energy and nutrients for growth, division, and survival. In recent years, fatty acid metabolism has received more and more attention considering it plays a key role in tumor progression (2). Several enzymes are involved in fatty acid metabolism, including fatty acid desaturase (FADS), fatty acid synthase, and carnitine acyltransferase 1. Of them, FADS family members (FADSs) play a crucial role in maintaining the dynamic balance of fatty acids *in vivo*. In mammals, FADSs include FADS1, FADS2, FADS3, FADS4 (SCD5), FADS5 (SCD), FADS6, FADS7 (DEGS1), and FADS8 (DEGS2) (3). The amino acid sequences of all FADSs contain three conservative motifs (HX3H, HX2HH, and HX2HHXFP), and they are mainly responsible for lipid conversion to regulate the balance of lipid metabolism.

FADSs are closely associated with cancer occurrence and development. For example, in comparison with normal tissues, FADS1 expression was found to be downregulated in non-small-cell lung cancer and bladder cancer tissues (4). Furthermore, low FADS1 expression was significantly associated with tumor location, size, and histological grade and predicted unfavorable prognosis in patients with cancer (4–6). Li et al. found FADS2 to be aberrantly expressed in, for example, BC, melanoma, lung cancer, brain cancer, liver cancer, colon cancer, and esophageal adenocarcinoma; moreover, it was found to play a key role in the growth, and progression, abnormal metabolism, and iron-dependent cell death of malignant tumors. The abnormal expression of FADS2 was significantly related to poor prognosis in patients with cancer (7). FADS5 expression was found to be highly upregulated in many tumors, promoting tumor cell proliferation, migration, and growth (8). The dynamic balance of fatty acids is known to play a key role in the occurrence and development of BC, but the functions of FADSs in BC have not yet been systematically reported.

Here, we used several databases, such as Gene Expression Profiling Interactive Analysis (GEPIA), The Cancer Genome Atlas (TCGA), Kaplan–Meier Plotter, Search Tool for the Retrieval of Interacting Genes/Proteins (STRING), Database for Annotation, Visualization, and Integrated Discovery (DAVID), cBioPortal, and Tumor Immune Estimation Resource (TIMER) to investigate the expression levels of FADSs in BC and assessed their diagnostic/prognostic value, molecular functions, genetic variations, immune cell infiltration status, related transcription factors, and kinases. The association of FADSs with various clinical subtypes, clinical stages, lymph node metastasis status, copy number variation, DNA methylation, and miRNA in BC was also analyzed. After that, for data validation, we selected FADS2 to assess its expression in clinical samples. The effects of FADS2 on BC proliferation, invasion, and metastasis were analyzed by experiments. Our objective was to evaluate the potential function of FADSs in BC and to try to provide a theoretical basis for diagnosis, treatment, and prognosis of BC.

## Materials and methods

### Cell lines and cultures

The human BC cell lines MCF-7, MDA-MB-231, T47D and Hs578t were maintained in high glucose Dulbecco’s modified Eagle’s medium (4.5 g/L glucose, HyClone, USA). SKBR-3, BT474, ZR-75-1, HCC1937, HCC1806, and BT549 cells were maintained in RPMI-1640 medium (HyClone). Both media were supplemented with 10% fetal bovine serum (Biochannel, Nanjing, China), 100 U/mL penicillin, and 100 mg/mL streptomycin. All cells were kept in a CO_2_ incubator (SANYO MCO-175, Japan) at 37°C and 5% CO_2_.

### Patient tissue samples

In total, seven groups of BC and adjacent normal tissue samples were collected from patients with BC (**Table S1**) (Southwest Hospital Affiliated to Third Military Medical University, Chongqing, China). All tissue-related experiments were performed as per the guidelines recommended by the Ethics Approval Committee of the institute.

### RNA extraction and qRT-PCR

Total RNA was extracted from BC cells and BC/adjacent normal tissue samples using TRIzol Reagent (Servicebio, Wuhan, China). Approximately 1 μg of total RNA was reverse-transcribed into cDNA synthesis using a PrimeScript™ RT Reagent Kit with gDNA Eraser (Perfect Real Time), according to the manufacturer’s instructions (Takara, Dalian, China). qRT-PCR was performed using TB Green™ Premix Ex Taq II (TaKaRa) on a CFX Connect™ Real-Time PCR Detection System (Bio-Rad, Hercules, CA, USA). *β-Actin* (forward: CTCCTACCTGGCCTCGCTGT, reverse: GCTGTCACCTTCACCGTTCC) served as the internal control. Data were analyzed using the comparative C_t_ method (ΔΔC_t_) (9). The primer sequences of FADS2 were as follows: GACCACGGCAAGAACTCAAAG (forward) and GAGGGTAGGAATCCAGCCATT (reverse).

### Protein extraction and western blotting

Western blotting was performed as described previously (10). Briefly, to obtain total protein, cells or tissues were lysed with RIPA lysis buffer (Beyotime, Shanghai, China) containing phenylmethanesulfonyl fluoride protease inhibitor (Beyotime). Protein concentration was measured using a detergent compatible Bradford protein assay kit (Beyotime). In total, 20 μg protein was loaded onto a 10% denaturing SDS-PAGE gel, and protein bands were then transferred to a 0.2-μm PVDF membrane (Merck Millipore, Germany). The membrane was blocked in 3% bovine serum albumin for 1 h, and FADS2 rabbit polyclonal antibody (Abcam, ab232898, 1:1000 dilution), E-cadherin rabbit polyclonal antibody (Proteintech, 20874-1-AP, 1:5000 dilution), N-cadherin rabbit polyclonal antibody (Proteintech, 66219-1-Ig, 1:5000 dilution), Snail rabbit polyclonal antibody (Proteintech, 13099-1-AP, 1:500 dilution), GAPDH rabbit polyclonal antibody (Proteintech, 10494-1-AP, 1:20000 dilution), or β-Actin rabbit polyclonal antibody (Abcam, ab8227, 1:5000 dilution) was added, followed by incubation at 4°C. After overnight incubation, the membrane was incubated with rabbit (Epizyme, LF102, 1:5,000 dilution) secondary antibody. Finally, the proteins bands were detected using the ECL system (Beyotime Biotechnology, China). This experiment was performed at least three times.

### Gene silencing

Two RNAi sequences (FADS2-RNAi-175, CCGGCCACGGCAAG-AACTCAAAGATCTCGAGATCTTTGAGTTCTTGCCGTGGTTTTTG and FADS2-RNAi-176, CCGGCCACCTGTCTGTCTACAGAAACTCGAGTTTCTGTAGACAGACAGGTGGTTTTTG) targeting human FADS2 mRNA and a negative control CON055 (hU6-MCS-CMV-RFP) were purchased from Genechem, Shanghai, China. MDA-MB-231 cells were transfected with the lentiviral vectors according to manufacturer instructions.

### Cell proliferation assays

Cells were plated into 96-well plates (3 × 10^3^ cells/well) and after incubation for 24 h, 10 μL MTS solution was added into each well, followed by incubation at 37 °C for 1 h (11). A microplate reader was used to measure the absorbance of each well at 492 nm. A proliferation curve was constructed with time as the abscissa and OD492 value of each group as the ordinate. Three independent experiments were performed for quantification.

### Migration and invasion assays

For the migration assay, the upper chamber (8.0 μm pore size, 6.5 mm diameter inserts, Corning, USA) was not precoated with Matrigel (BD Biosciences, CA). Overall, 5 × 10^4^ BC cells suspended in serum-free medium were placed into the upper chambers. To the lower chambers, 600 μL medium mixed with 10% fetal bovine serum was added. After 48 h of incubation, the cells on the upper surface of the filter were removed using cotton swabs. Migrating cells on the lower surface were fixed in 4% paraformaldehyde and stained with 0.1% crystal violet. Finally, cells were counted in five random fields under a microscope (Nikon, Tokyo, Japan) at a magnification of 100×. To assess the invasion ability, transwelll assay was performed, for which the upper chamber was precoated with Matrigel. The experiment was performed at least three times.

### Data and visualization

Data concerning mRNA expression, DNA methylation, copy number variants, and clinical phenotype of FADSs in BC were obtained from an online website (https://xenabrowser.net/datapages/). Two datasets were collected from the Gene Expression Omnibus (GEO) database: GSE42568 (104 BC and 17 normal samples) and GSE65194 (153 BC and 11 normal samples). Receiver operating characteristic (ROC) curves were plotted using the R package “pROC”. Correlation results were visualized with the R package “ggpubr”.

### The human protein atlas database

The HPA database (www.proteinatlas.org) provides free access to immunohistochemistry results for many cancer tissues. Depending on staining intensity and percentage of stained cells, immunohistochemistry score is primarily divided into strong, medium, weak, and negative (12). In this study, protein expression levels of FADSs in BC and normal breast tissues were analyzed using the “Pathology Atlas” and “Tissue Atlas” modules on www.proteinatlas.org.

### Kaplan–Meier plotter database

We used the Kaplan–Meier plotter database (http://kmplot.com/) to assess the impact of 54,000 human genes on the prognosis of various cancers, such as BC (n = 6234), ovarian cancer (n = 2190), lung cancer (n = 3452), and gastric cancer (n = 1440) (13). The effects of FADSs on overall survival (OS) and recurrence-free survival (RFS) of patients with BC were determined according to the median gene expression value. *P* < 0.05 indicated a statistically significant difference.

### cBioportal

cBioportal (https://www.cbioportal.org/), an online open-access website containing 225 cancer studies, is widely used for exploring, visualizing, and analyzing multidimensional cancer genomics data (14, 15). Herein we used the “TCGA, PanCancer Atlas” dataset (including 994 complete case reports) to explore genetic alterations in FADSs in BC.

### STRING and DAVID

STRING (https://string-db.org/) is an online database that aims to collect and integrate all publicly available protein–protein interaction (PPI) information (16). We used this database to construct PPI networks with FADSs; the interaction score was > 0.4. Cytoscape 3.7.2 was used for data visualization. Gene ontology (GO) and Kyoto Encyclopedia of Genes and Genomes (KEGG) pathway enrichment analyses were performed with DAVID (https://david.ncifcrf.gov/) (17).

### TRRUST v2

TRRUST v2 (www.grnpedia.org/trrust), a freely available and manually curated database, contains 8,444 transcription factor–target regulatory relationships of 800 human transcription factors (18). We herein analyzed the transcription factors of FADSs using the “find key regulators for query genes” module of this database.

### LinkedOmics

LinkedOmics (http://www.linkedomics.org) includes multi-omics and clinical data for 32 cancer types and 11,158 patients from TCGA project (19). We explored the kinase targets of FADSs based on the “RNA-seq” dataset of the BI Institute.

### Tumor immune estimation resource

The TIMER database (https://cistrome.shinyapps.io/timer/) was used for analyzing the infiltration of immune cells in tumor tissues based on the RNA-seq expression profile data in TCGA (20). Using the “gene” module of this database, we explored the association between FADSs and infiltration abundance of six tumor-infiltrating immune cells.

### Statistical analysis

Unpaired *t*-test was used to analyze differences in transcription levels. Survival analysis was performed using the log-rank test. All statistical analyses were performed on SPSS v22 (IBM). GraphPad Prism was used for plotting charts and graphs. *P* ≤ 0.05 indicated statistical significance.

## Results

### Correlation between various clinical factors and expression of FADSs in BC

**Figure 1:**
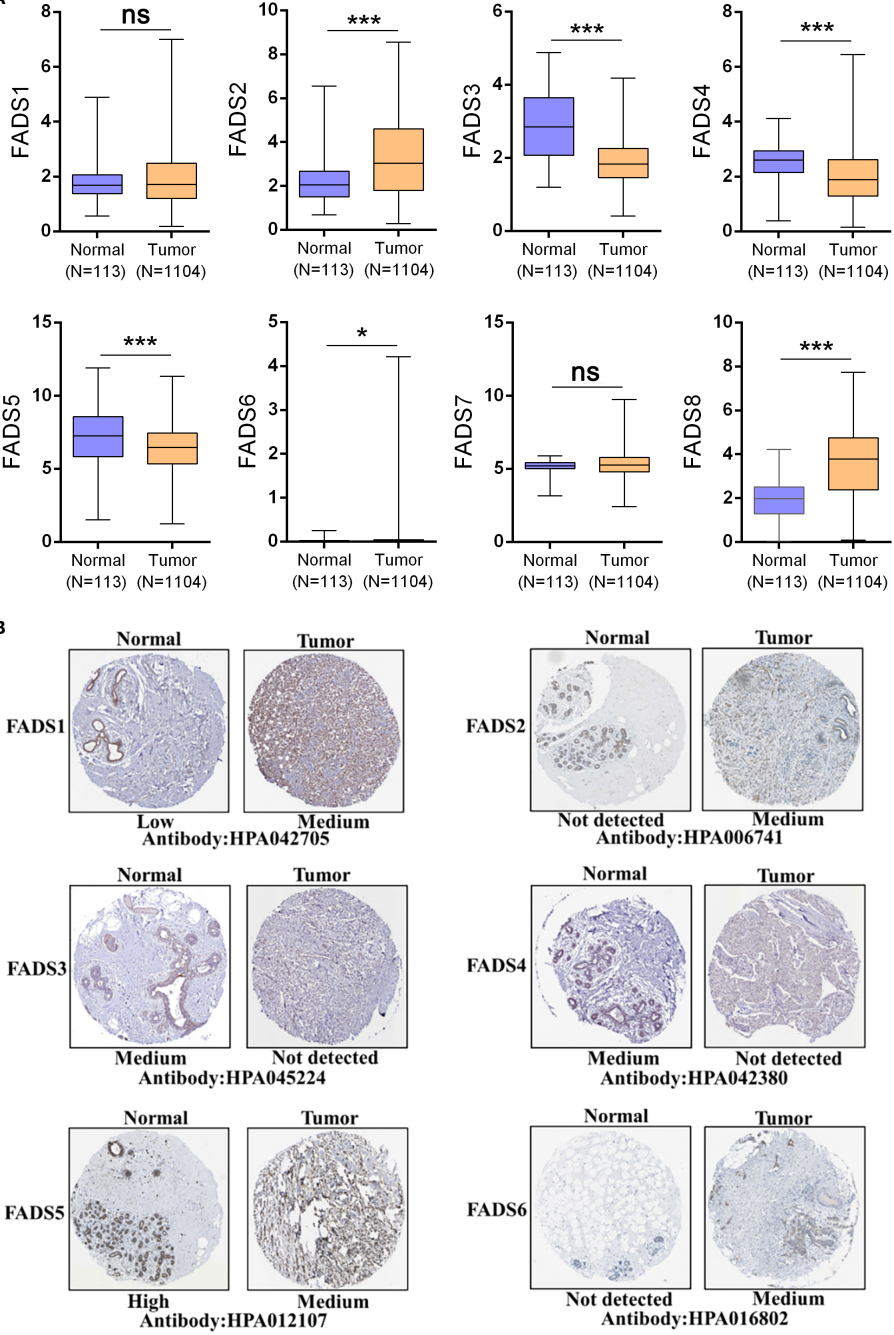
The expression levels of FADSs in BC. **(A)** mRNA expression levels of FADSs in BC and normal breast tissues, as plotted using GraphPad Prism (^ns^ *P* > 0.05, ^*^ *P* < 0.05, ^***^ *P* < 0.001). **(B)** Protein expression levels of FADSs in BC and normal breast tissues in the HPA database.

FADSs are responsible for lipid conversion; the main differences between them are their choice of lipid substrates and sites of action (**Table S2**). We studied the expression levels of FADSs in BC based on data from the TCGA cohort. The transcriptional levels of FADS2/6/8 were significantly elevated (*P* < 0.001), while those of FADS3/4/5 were significantly reduced. Besides, the transcriptional levels of FADS1/7 did not show a considerable change between BC and normal breast tissues (**Figure 1A**). We also investigated the protein expression levels of FADSs in BC based on the HPA database (**Figure 1B**) and found that the protein expression levels of FADS1/2/6 were higher in BC tissues than in normal breast tissues, while those of FADS3/4/5 were lower in the former than in the latter. The protein expression level data were almost consistent with transcriptional level data of FADSs. Notably, immunohistochemical data for FADS7/8 was not present in the HPA database.

**Figure 2:**
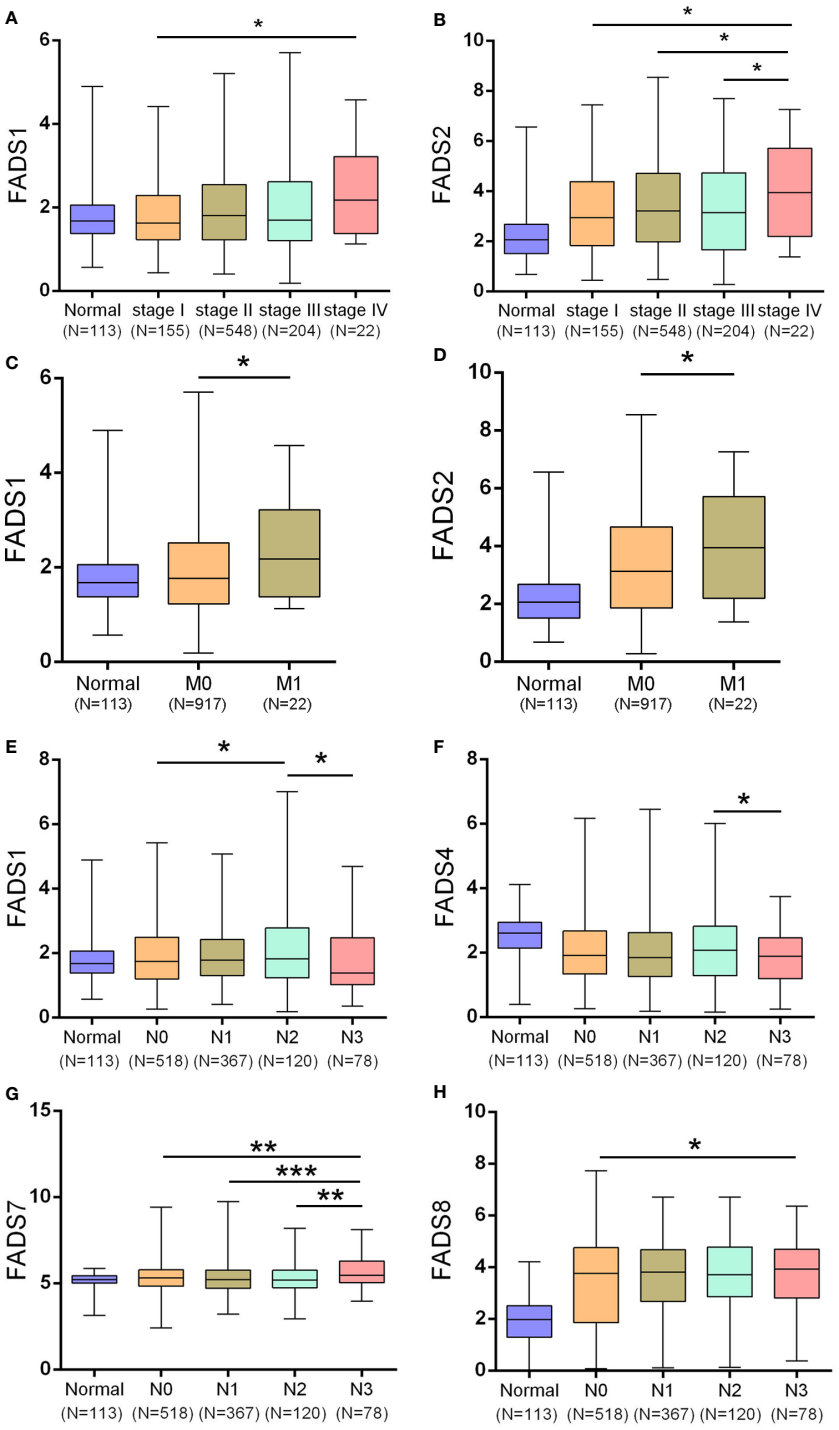
Correlation between various clinical factors and expression of FADSs in BC. **(A, B)** The mRNA expression levels of FADS1/2 in different breast cancer clinical stages, respectively. **(C, D)** The mRNA expression levels of FADS1/2 in different breast cancer M stages, respectively. **(E–H)** The mRNA expression levels of FADS1/4/7/8 in different breast cancer N stages (lymph node metastasis in BC), respectively. ^*^ *P* < 0.05, ^**^ *P* < 0.01, ^***^ *P* < 0.001.

To further assess the correlation between the expression of FADSs and clinical parameters, we investigated their expression levels in various clinical stages, subtypes, and lymph node metastasis status based on TCGA. We found that FADS1/2 expression was significantly upregulated in stage IV BC (**Figures 2A, B**). The expression levels of other FADSs showed no significant association with pathological grade. These findings indicated that the expression of FADS1/2 in BC may be related to tumor progression.

High expression levels of FADS1 (**Figure 2C**) and FADS2 (**Figure 2D**) were significantly correlated with the M classification of BC (*P* = 0.0375 for FADS1 and *P* = 0.0381 for FADS2), implying that FADS1/2 expression levels are significantly related to distant metastasis of BC.

We also determined the correlation between mRNA expression levels of FADSs and lymph node metastasis in BC and found that FADS1 expression was significantly lower in patients with BC classified as N3 (**Figure 2E**; N3, metastases in ≥10 axillary lymph nodes) than in those classified as N2 (N2, metastases in 4–9 axillary lymph nodes). Further, FADS4 expression was lower in patients with BC classified as N3 (**Figure 2F**). FADS7 expression was considerably increased in patients with BC classified as N3 than in those classified as N0, N1, and N2 (**Figure 2G**; N1, metastases in 1–3 axillary lymph nodes), and the FADS8 expression level was upregulated in patients with BC classified as N2 and N3 than in those classified as N0 (N0, no regional lymph node metastasis; **Figure 2H**). Altogether, these results suggested a close correlation between FADS1/4/7/8 expression and lymph node metastasis in BC.

**Figure 3:**
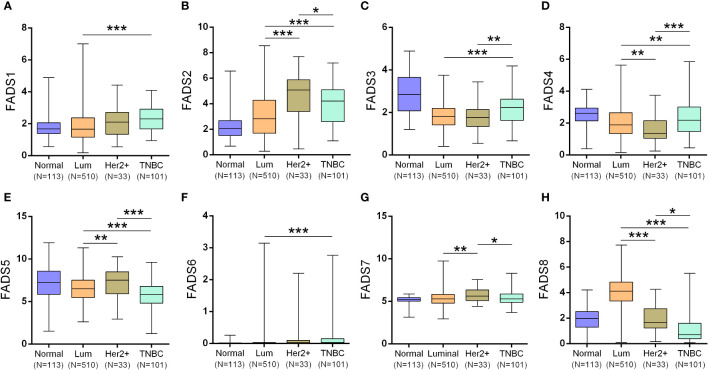
Box plots showing the expression of FADSs in different BC subtypes (luminal BC, HER2-positive BC, and TNBC) (^*^ *P* < 0.05, ^**^ *P* < 0.01, ^***^ *P* < 0.001). FADS1 **(A)**, FADS2 **(B)**, FADS3 **(C)**, FADS4 **(D)**, FADS5 **(E)**, FADS6 **(F)**, FADS7 **(G)**, FADS8 **(H)** expression levels in different breast cancer subtypes.

Neither of the three subtypes showed any significant changes in FADS1 expression (**Figure 3A**). FADS2/5 expression (**Figures 3B–E**) was significantly upregulated and FADS4 expression (**Figure 3D**) was significantly downregulated in HER2-positive BC than those in luminal BC and triple-negative BC (TNBC). FADS3 expression was significantly upregulated in TNBC than in luminal BC and HER2-positive BC (**Figure 3C**). FADS6 expression was low in all BC subtypes (**Figure 3F**). Besides, FADS7 expression was markedly downregulated in patients with HER2-positive BC (**Figure 3G**). A significant increase in FADS8 expression was exclusively observed in luminal BC (**Figure 3H**).

Finally, the mRNA expression levels of FADSs in BC were validated in two GEO datasets (GSE42568 and GSE65194; **Figure S1**). The expression levels of FADS2/3/5/8 in these GEO datasets were highly consistent with those in the TCGA database (**Figure 1A**). However, the expression levels of FADS4/7 in only GSE65194 dataset were consistent with those in the TCGA database. Unfortunately, data about the expression levels of FADS1/6 were not present in either GSE42568 or GSE65194. **Table S3** summarizes the comparison of the expression levels of FADSs in TCGA, GSE42568 and GSE65194 databases.

### Potential of FADSs to diagnose BC

**Figure 4:**
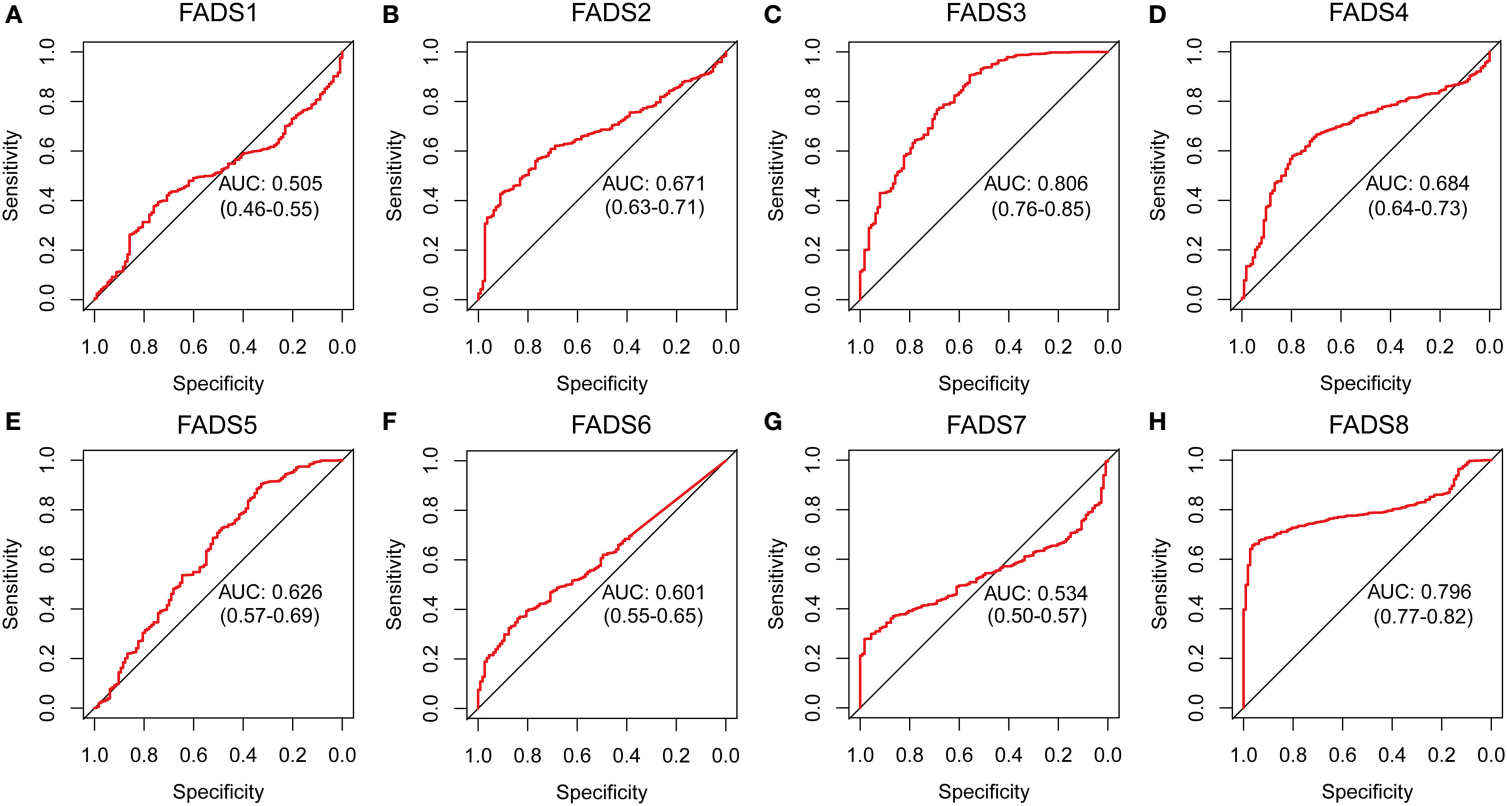
ROC analysis to assess the sensitivity and specificity of FADSs to diagnose BC. AUC, area under curve. **(A–H)** Receiver operating characteristic curve of FADSs in diagnosis of breast cancer.

The ROC curve is typically used to evaluate clinical utility for both diagnostic and prognostic models (21). Area under the ROC curve (AUC) values of close to 1 are indicative of robust overall diagnostic performance. To explore the potential of the expression of FADSs to diagnose BC, we downloaded a BC dataset from TCGA, that contains 1104 BC and 113 normal tissue samples, from UCSC (https://xenabrowser.net/datapages/). ROC analysis was performed according to the mRNA expression level of FADSs in BC and normal tissue samples. The AUC of FADS2/3/4/8 was found to be >0.65 (**Figure 4**), and they had better ability to distinguish between patients with and without BC. Thus, we believe that FADS2/3/4/8 could serve as reliable diagnostic markers for BC.

### Genetic alterations and correlation between expression, DNA methylation, copy number variants and miRNA regulation of FADSs in BC

**Figure 5:**
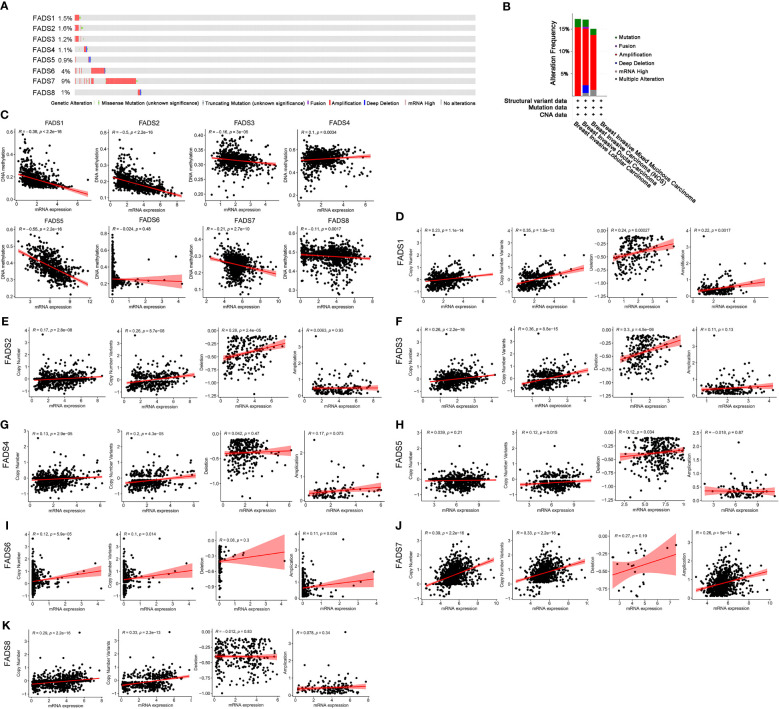
Genetic alterations and correlation between expression, DNA methylation, and copy number variants of FADSs in BC. **(C)** Pearson correlation test showing the relationship between expression of FADSs and DNA methylation. **(D–K)** Correlation analysis between mRNA expression levels of FADSs and copy number variants.

Gene expression levels are affected by various factors, such as copy number variants, DNA methylation, and histone modification. The expression of FADSs was found to be altered in 166 samples of 996 patients with BC (17%). The expression of eight FADSs was altered in 1.5%, 1.6%, 1.2%, 1.1%, 0.9%, 4%, 9%, and 1% of the queried BC samples, respectively (**Figures 5A, B**). To elucidate the reason for differences in the expression of FADSs in BC, we analyzed the association between the mRNA expression level of FADSs and DNA methylation (**Figure 5C**) and copy number variants (**Figures 5D–K**). The mRNA expression level of all FADSs, except FADS6, was observed to be significantly negatively correlated with DNA methylation. Furthermore, concerning copy number variants, Pearson correlation test indicated that FADS1 mRNA expression was related to both copy number amplification and deletion. FADS2/3/5 and FADS6/7 mRNA expression levels were positively correlated with copy number deletion and amplification, respectively. Although FADS5/8 mRNA expression levels were found to have a significant correlation with copy number variants, there was no correlation with amplification or deletion (**Table S4**). Furthermore, the miRNAs that target and regulate the expression of FADSs were predicted by ENCORI (https://starbase.sysu.edu.cn/), and the correlation between the expression levels of FADSs and miRNAs, as well as the expression of miRNAs in BC were analyzed (**Table S5**). Except for FADS6, which was not found in ENCORI, other FADS-targeting miRNAs were significantly correlated with the expression of FADSs. These findings suggest that copy number variants, miRNA regulation and/or DNA methylation influence the expression of FADSs in BC.

### Prognostic value of FADSs in patients with BC

**Figure 6:**
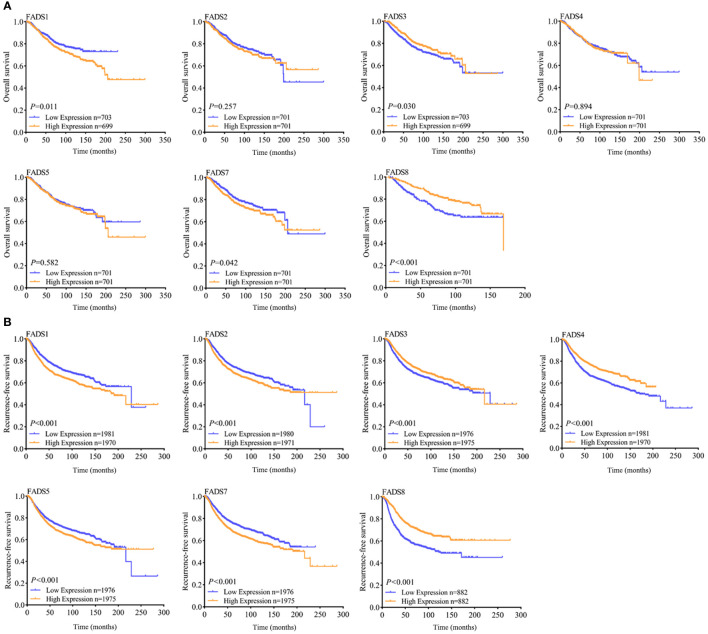
OS **(A)** and RFS **(B)** curves indicating the prognostic value of FADSs in BC (Kaplan–Meier plotter database).

To determine the correlation between differentially expressed FADSs and prognosis, the Kaplan–Meier plotter database was used to analyze the effects of FADSs on the OS (**Figure 6A**) and RFS (**Figure 6B**) of patients with BC. Higher mRNA expression levels of FADS1 (**Figure 6A**, *P* = 0.011) and FADS7 (**Figure 6A**, *P* = 0.042) were significantly correlated with short OS, while lower mRNA expression levels of FADS3 and FADS8 were significantly correlated with short OS (**Figure 6A**). The mRNA expression level of other FADSs did not seem to significantly affect OS. Further, higher FADS1/2/5/7 mRNA expression levels were correlated with poor RFS (**Figure 6B**; *P* < 0.001), while lower FADS3/4/8 mRNA expression levels showed better prognosis in terms of RFS (**Figure 6B**; *P* < 0.001).

### Transcription factors and kinases of FADSs

Based on TRRUST v2, we found that FADS4 and FADS5 are regulated by SREBF1 (*P* < 0.001, **Table S6**). The correlation between FADS4/5 and SREBF1 in BC was analyzed using TIMER (**Figure S2**). The mRNA expression level of SREBF1 was significantly negatively FADS4 (r = -0.219, *P <* 0.001), and positively associated with FADS5 (r = 0.502, *P* < 0.001).

We then analyzed kinases related to FADSs using LinkedOmics and found CDK1, CDK2, and CSNK2A1 to be related to FADS1/2; PRKCA, PRKCD, ATM, CDK1, and CDK2 to FADS3; PRKCA to FADS4; CDK1 and CSNK2A1 to FADS5; CDK1 and CDK2 to FADS6; and CDK2, CSNK2A1, CHEK1, and ATM to FADS7/8. FADS7 also interacted with CDK1 (**Table S7**).

### Gene set enrichment analysis of FADSs

**Figure 7:**
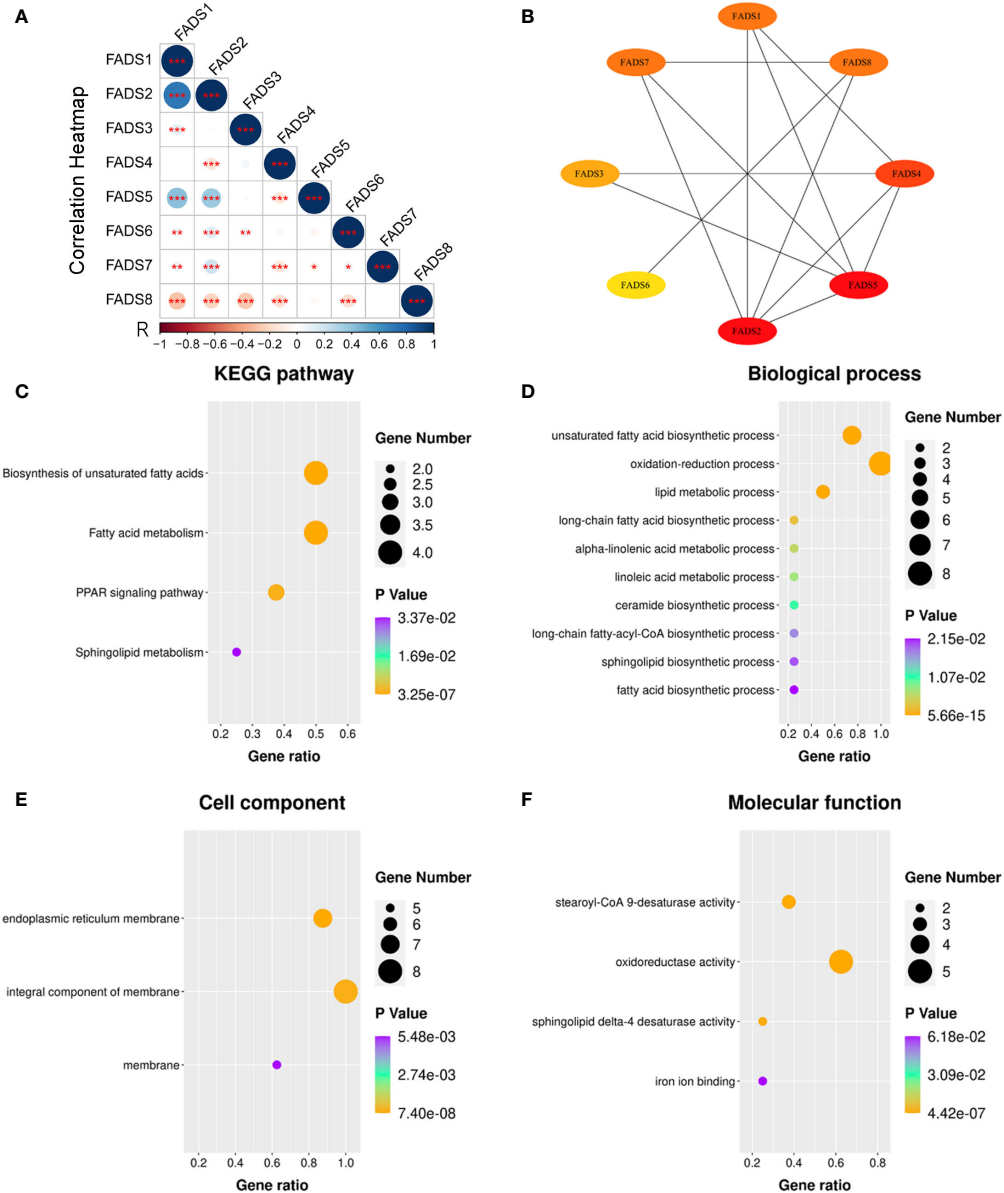
PPI network and GO and KEGG pathway enrichment analyses for FADSs in BC were conducted using STRING and DAVID, respectively. **(A)** A heat map showing the correlation of eight FADSs in BC (^*^ *P* < 0.05, ^**^ *P* < 0.01, ^***^ *P* < 0.001). **(B)** PPI network. **(C)** KEGG pathway enrichment analysis. **(D)** Terms enriched in the biological process, **(E)** cellular component, and **(F)** molecular function categories in BC.

The summary of the correlation between FADSs in BC was analyzed using cBioportal. Moreover, the Pearson correlation was calculated by analyzing the mRNA expression levels of FADSs (**Figure 7A**). FADS1 was significantly strongly associated with FADS2 (r = 0.759), and there was moderate correlation between FADS5 and FADS1 (r = 0.401) and FADS2 (r = 0.403).

To further explore the possible biological functions of FADSs, we constructed a PPI network and performed functional enrichment analyses. The PPI network showed eight nodes and 13 edges (**Figure 7B**). KEGG pathway enrichment analysis suggested the involvement of FADSs in the biosynthesis of unsaturated fatty acids, fatty acid metabolism, peroxisome proliferator-activated receptor (PPAR) signaling pathway, and sphingolipid metabolism (**Figure 7C**). GO enrichment analysis indicated that in the category of biological processes, FADSs were mainly associated with fatty acid metabolism (e.g., unsaturated fatty acid biosynthesis, oxidation–reduction process, and lipid metabolic process; **Figure 7D**), and in the category of cellular component, they were distributed in organelle membranes, such as endoplasmic reticulum membrane (**Figure 7E**). Further, in the category of molecular function, FADSs were involved in stearoyl-CoA 9-desaturase activity, oxidoreductase activity, sphingolipid delta-4 desaturase activity, and iron ion binding (**Figure 7F**).

### Association between FADSs and tumor-infiltrating immune cells in BC

**Figure 8:**
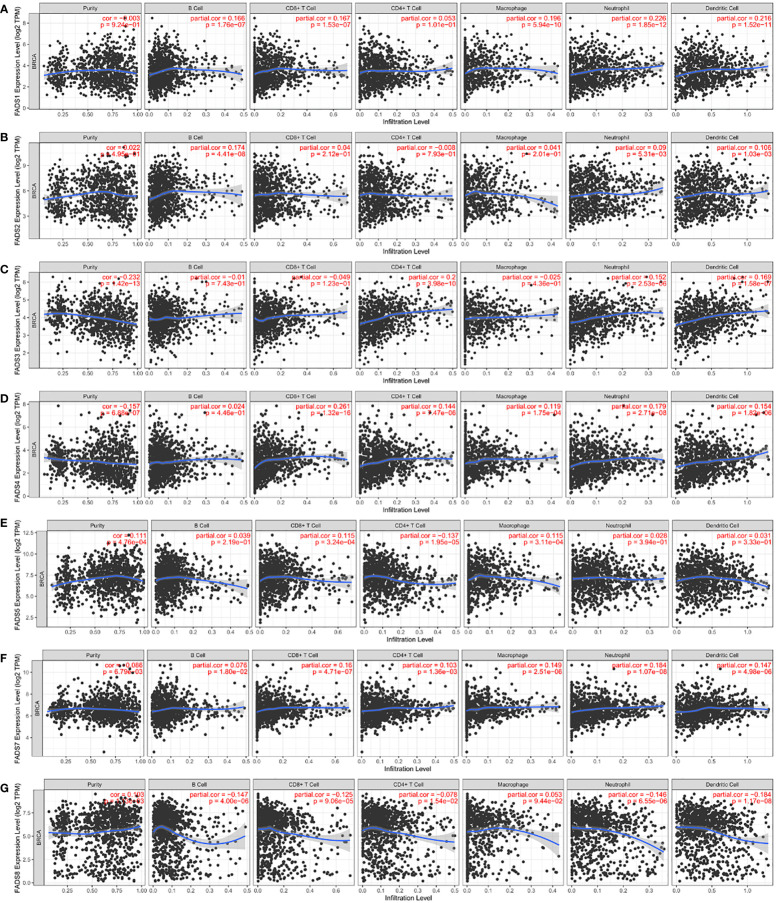
Correlation analysis between FADSs and tumor-infiltrating immune cells. Correlation analysis of FADS1 **(A)**, FADS2 **(B)**, FADS3 **(C)**, FADS4 **(D)**, FADS5 **(E)**, FADST7 **(F)**, and FADS8 **(G)** with tumor-infiltrating immune cells.

Gene set enrichment analysis revealed an association between FADSs and lipid metabolism processes. Many studies have revealed that changes in lipid metabolism have critical effects on the fate, proliferation, and function of immune cells (22). In this study, we used the TIMER database to investigate the association between FADSs and tumor-infiltrating immune cells, including B cells, CD4+ T cells, CD8+ T cells, neutrophils, macrophages, and dendritic cells. We found that FADS1 expression levels had a significant positive correlation with the infiltration of B cells (r = 0.166, *P* < 0.001), CD8+ T cells (r = 0.167, *P* < 0.001), macrophages (r = 0.196, *P* < 0.001), neutrophils (r = 0.226, *P* < 0.001), and dendritic cells (r = 0.216, *P* < 0.001) and no significant correlation with CD4+ T cells in BC (**Figure 8A**). Further, FADS2 expression levels were positively correlated with the infiltration of B cells (r = 0.174, *P* < 0.001), neutrophils (r = 0.09, *P* < 0.01), and dendritic cells (r = 0.106, *P* < 0.01) (**Figure 8B**), and FADS3 expression levels were positively correlated with the infiltration of neutrophils (r = 0.152, *P* < 0.001), dendritic cells (r = 0.169, *P* < 0.001), and CD4+ T cells (r = 0.2, *P* < 0.001) (**Figure 8C**). FADS4 expression was positively associated with the infiltration of CD8+ T cells (r = 0.261, *P* < 0.001), CD4+ T cells (r = 0.144, *P* < 0.001), macrophages (r = 0.119, *P* < 0.001), neutrophils (r = 0.179, *P* < 0.001), and dendritic cells (r = 0.154, *P* < 0.001) (**Figure 8D**), and FADS5 expression was positively correlated with the infiltration of CD8+ T cells (r = 0.115, *P* < 0.001) and macrophages (r = 0.115, *P* < 0.001) but negatively correlated with that of CD4+ T cells (r = -0.137, *P* < 0.001) (**Figure 8E**). FADS7 expression level was positively and significantly associated with the infiltration of all six types of immune cells in BC (**Figure 8F**). FADS8 expression, on the other hand, was negatively correlated with the infiltration of all immune cell types, except macrophages (**Figure 8G**; **Table S8**).

### Functional validation of FADS2

**Figure 9:**
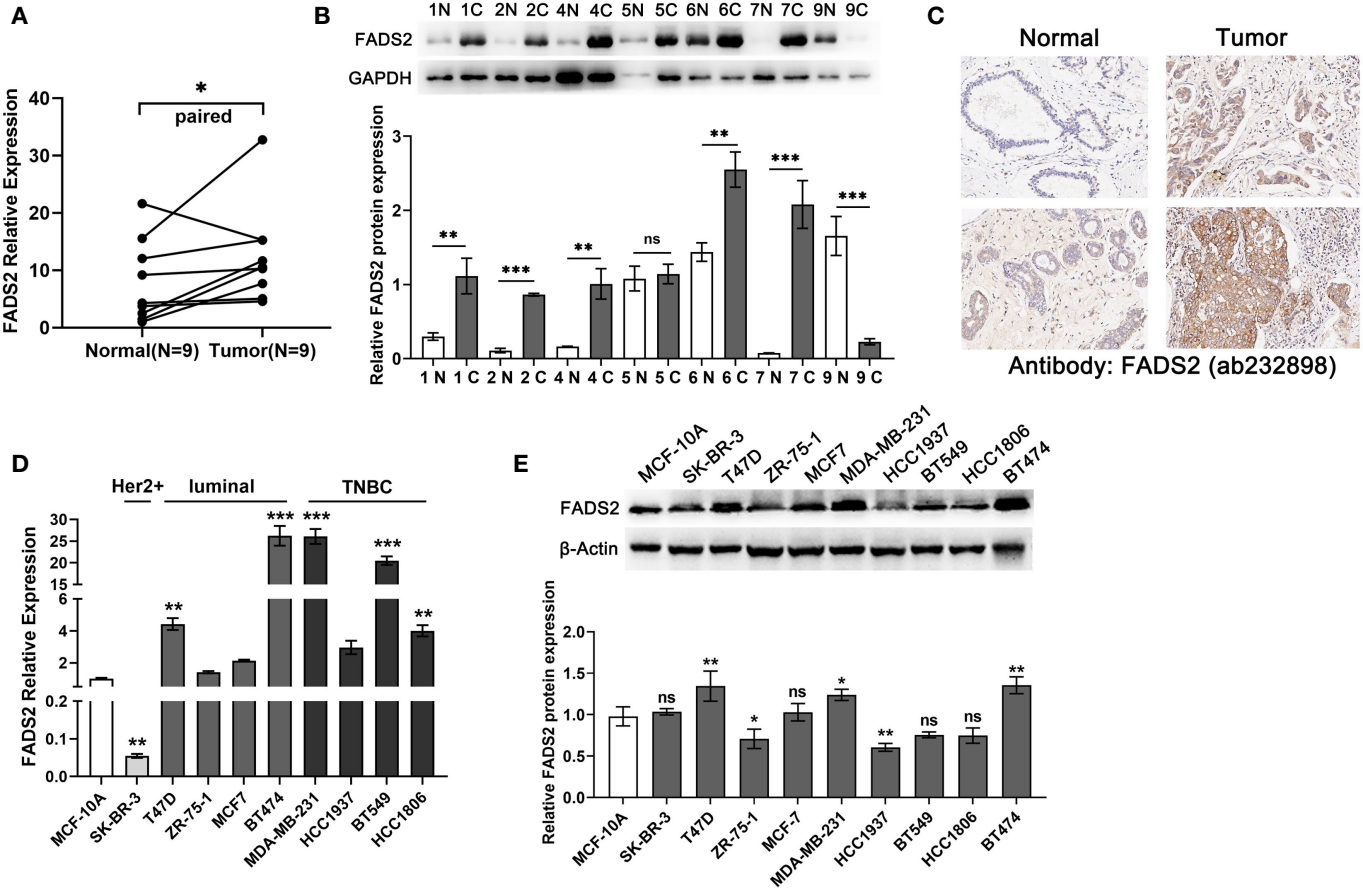
The expression of FADS2 in BC. **(A, B)** RT-qPCR and Western blotting to detect the expression of FADS2 in breast cancer clinical tissue samples, GAPDH acts as control. N: normal, C: Tumor. **(C)** Immunohistochemical analysis of FADS2 expression in breast cancer tissues and adjacent tissues. **(D, E)** RT-qPCR and Western blotting to detect the expression of FADS2 in breast cancer cell lines, β-actin acts as control. ^ns^ *P* > 0.05, ^*^ *P* < 0.05, ^**^ *P* < 0.01, ^***^ *P* < 0.001.

The above results indicated that FADS2 is related to the prognosis of BC and may be an important prognostic marker. We further tested whether the FADS2 signatures were independent prognostic factors by constructing a model related to the prognosis of BC patients. LASSO Cox regression analysis and multivariate Cox regression analysis were carried out sequentially and seven FADS2-associated genes that significantly correlated with the prognosis of BC patients were identified as hub genes to construct the prognostic risk model. We examined the roles of the seven FADS2-related gene signatures using the testing cohort GSE20685 (n = 327). The results suggested that FADS2 was significantly related to the prognosis of patients with BC (**Figure S3**). Therefore, FADS2 was identified as a prognostic marker in BC and subjected to further exploration. We validated its expression level in BC clinical tissue samples and cell lines and found that in comparison with adjacent tumor samples, FADS2 was highly expressed in BC tissue samples (**Figures 9A, B**). We further validated our predictions using immunohistochemistry (IHC) and found that FADS2 was upregulated in the BC tissue samples (**Figure 9C**). Compared with normal breast cell line MCF-10A, FADS2 was highly expressed in cell lines T47D, MDA-MB-231, and BT474 (**Figures 9D, E**).

**Figure 10:**
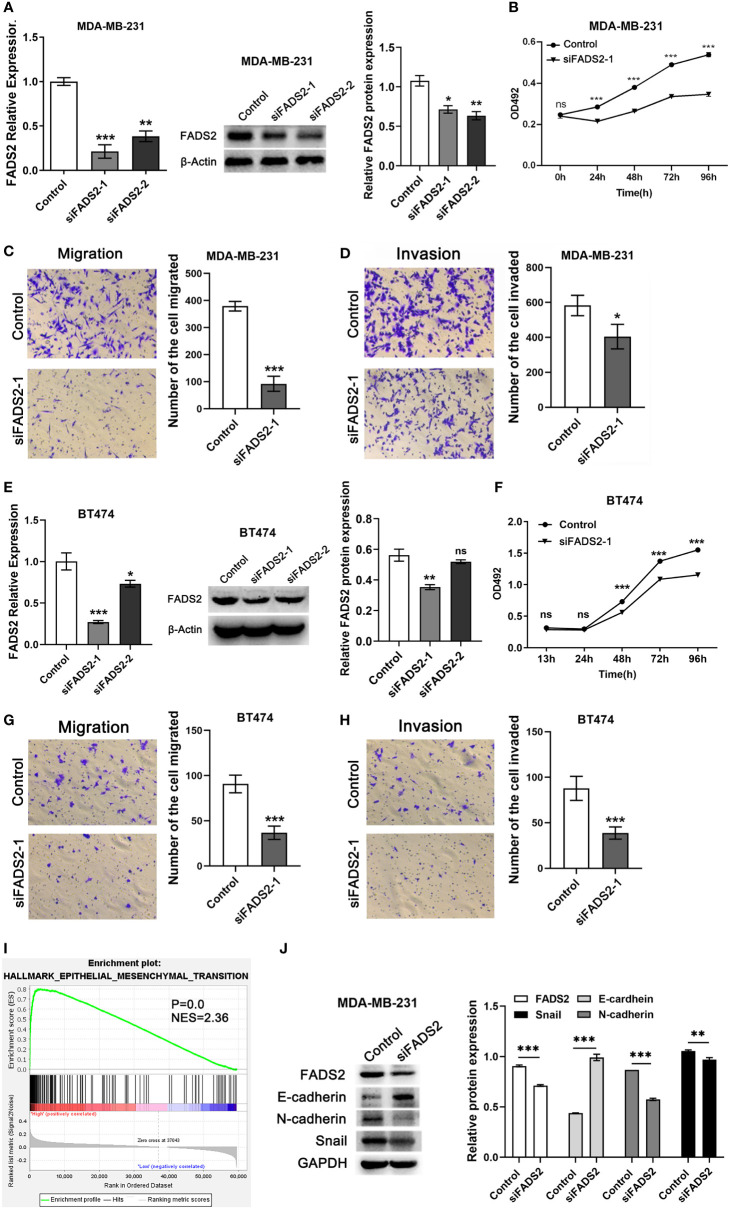
Effects of FADS2 knockdown on the functions of BC cell lines. **(A–E)** RT-qPCR and Western blotting to detect the expression of FADS2 in MDA-MB-231 and BT474 cells, β-actin acts as control. **(B–F)** MTS assay was used to detect the effect of FADS2 down-regulation on cell proliferation. **(C–G)** Transwell cell migration assay to detect the effect of FADS2 down-regulation on cell migration ability. **(D–H)** Transwell cell invasion assay was used to detect the effect of FADS2 down-regulation on cell invasion ability. **(I)** Enrichment of EMT signaling pathway with FADS2 expression was shown using enrichment analysis of TCGA. **(J)** Relative expression levels of FADS2, E-cadherin, N-cadherin and Snail in the MDA-MB-231 cells transfected with Control or siFADS2, GAPDH acts as control. ^ns^ *P* > 0.05, ^*^ *P* < 0.05, ^**^ *P* < 0.01, ^***^ *P* < 0.001.

Further, to validate the function of FADS2 in BC, we performed functional experiments after FADS2 was knocked down by siRNAs in MDA-MB-231 and BT474 cells. qRT-PCR and Western blotting were performed to examine the efficiency of knockdown (**Figures 10A–E**). We found that FADS2 knockdown decreased cell viability (**Figures 10B–F**); in addition, transwell assay results indicated that the migration (**Figures 10C–G**) and invasion abilities of BC cells were decreased after FADS2 knockdown (**Figures 10D–H**). In further exploration of the mechanism by which FADS2 promotes invasion and migration of BC, Gene set enrichment analysis (GSEA) analysis showed that FADS2 expression was related to EMT signaling pathway (P = 0.0, NES = 2.36, **Figure 10I**). Western blotting was used to analyze EMT markers induced by FADS2 in the MDA-MB-231 cell lines. The protein level of the epithelial marker E-cadherin was notably increased while the expression of mesenchymal markers, such as N-cadherin and Snail were considerably decreased in FADS2 knockdown cells (**Figure 10J**).

## Discussion

FADSs play a pivotal role in the occurrence and development of various types of tumors (4–7). However, their role in BC remains unclear. Herein we systematically analyzed mRNA and protein expression levels, genetic alterations, copy number variants, DNA methylation, and potential functions of FADSs in BC. We also studied the relationship between the expression of FADSs and clinicopathological parameters, diagnosis, prognosis, and tumor-infiltrating immune cells. FADS2 was found to be highly expressed in BC, and the role of FADS2 in BC was evaluated by clinical sample validation and functional experiments. Our findings suggested that FADS1/2/3/4 could serve as prognostic markers, providing novel options for the early diagnosis and treatment of patients with BC.

The expression levels of FADS1/2 in BC tissues were found to be higher than those in normal breast tissues, while the expression levels of FADS3/4 were lower in BC tissues than those in normal breast tissues. Among them, the mRNA expression level of FADS1 was not significantly changed between BC and normal breast tissue, but the protein expression level was significantly increased, possibly because of regulation by post-translational modification. Our data validated that differential expression levels of FADSs are related to poor prognosis in patients with BC. More importantly, FADS2/4 expression was found to be correlated with clinical grade. Previous studies have reported that FADSs play diverse roles in various types of cancers. For example, Zhao et al. found that FADS1 was highly expressed in esophageal squamous cell carcinoma and it regulated cell proliferation, migration, and invasion *via* the Akt/mTOR pathway (23). Triki et al. reported that FADS2 expression was associated with the mTOR signal transduction pathway and SREBP-1 activity in mammals (24). The activated mTOR signal transduction pathway promoted the transformation of palmitic acid into saponins by upregulating FADS2 expression. In addition, Han et al. found FADS3 expression levels to be lower in lung adenocarcinoma tissues than in normal tissues; further, its expression was significantly related to immune B cell infiltration and prognosis in patients with lung adenocarcinoma (25). FADS4 is a Δ9-desaturase in many vertebrates and is found in the fetal brain, adult brain, and pancreas. The ratio of monounsaturated to saturated fatty acids *in vivo* is determined by FADS4 activity. FADS4 is associated with cell proliferation and differentiation regulation, signal transduction, and organ development (26). Collectively, our data suggest that FADS1/2/3/4 play a crucial role in BC; further investigations are warranted to understand their role in BC even more comprehensively.

Although we generated a limited amount of data for FADS5/6/7/8, it is notable that even they play a chief role in tumor occurrence and development in humans. Of them, FADS5 and FADS4 have similar functions and represent a class of widely expressed fatty acid Δ9-desaturase; they regulate the transformation of saturated fatty acids into monounsaturated fatty acids and also play a central regulatory role in cell proliferation, differentiation, and survival (27). FADS6 is a key bifunctional enzyme that regulates the biosynthesis of polyunsaturated fatty acids *via* the ω6 and ω3 pathways to desaturate linoleic/α-linolenic acid (28). ABTL0812, a small molecule compound with anticancer activity, can inhibit FADS7 activity to increase long-chain dihydroceramide levels in cells, leading to persistent endoplasmic reticulum stress. Besides, ABTL0812 activates the unfolded protein reaction *via* ATF4–DDIT3–TRIB3 and eventually promotes the cytotoxic autophagy of cancer cells (28). Altogether, these findings suggest that FADS7 is involved in cancer occurrence and development. Similarly, FADS8 expression has been reported to be upregulated in BC (29), and FADS7/8 blocking by siRNA was found to indirectly enhance dihydroceramide activity and reduce cell proliferation (30). To date, no direct studies exist on these FADSs in cancer. Collectively, the results reported herein and those of previous studies suggest that FADSs are directly or indirectly associated with tumor occurrence and progression; they thus have high research and exploratory significance.

The results of functional enrichment analyses showed that FADSs are principally involved in fatty acid metabolism, PPAR signaling pathway, and sphingolipid metabolism. Lipid metabolism reprogramming is a major feature of breast cancer cells and abnormal lipid metabolism is related to the occurrence and development of BC. Breast cancer cells use lipid metabolism to obtain energy, biomembrane components, and signal molecules needed for cancer cell proliferation, survival, invasion, metastasis, response to the tumor microenvironment, and cancer treatment (31). At the same time, lipid metabolism reprogramming changes the carcinogenic signaling pathway in BC cells (32). Research showed that there are significant differences in lipid metabolism in different types of BC cell. For example, compared with MCF-7, MDA-MB-231 cells have a higher percentage of saturated fatty acids, fatty acids and polyunsaturated fatty acids (PUFA), while MCF-7 cells have a higher percentage of monounsaturated fatty acids (33). In addition, different types of lipids have different regulatory effects on breast cancer. Omega 3-docosahexaenoic acid, a fatty acid, decreased proliferation and invasive potential, and increased apoptosis of the human breast cells (34, 35). However, studies have demonstrated that a diet rich in ω-6 PUFAs has strong promoting effects on BC development (36). Therefore, it is of great significance to explore the impact of lipid metabolism reprogramming on the progression and therapeutic response of BC, to find potential new targets for diagnosis and treatment. PPARs are ligand-activated transcription factors belonging to the nuclear hormone receptor superfamily and including three major members: PPAR*α*(or NR1C1), PPAR*β*/*δ* (or NR1C2), and PPAR*γ* (or NR1C3). The transcriptional activity of PPARs can be regulated by non-genomic effects with phosphatases and kinases, including ERK1/2, P38-MAPK, PKA, PKC, AMPK, and GSK3 (37). Furthermore, PPARs reportedly form heterodimers with retinoic acid X receptors; regulate the expression of genes involved in lipid metabolism, lipogenesis, metabolic homeostasis maintenance, and inflammation; and induce anticancer effects in a variety of tumors (37, 38). Some research showed that the PPAR signaling pathway plays an important role in promoting the occurrence and development of BC (38–40). In addition, Chen et al. showed that the PPAR pathway may be an important predictor of genes involved in chemotherapy response in BC (41). Interestingly, PPARα can up-regulate the promoter activity of FADS2 in fish (42). A PPARδ agonist used in treating pancreatic cancer increases the expression of FADS2 at the mRNA and protein levels (43). The inhibition of PPARγ significantly attenuated the upregulation of FADS5 and slightly decreased the upregulation of FADS2 (44). Furthermore, Reardon et al. found that the PPARγ antagonist GW9662 prevented FADS3 upregulation in human liver-derived HepG2 cells (45). Although the relationship between PPAR pathway and FADSs has not been reported in BC, our data and previous studies indicate that FADSs may affect the lipid metabolism of tumor cells through the PPAR signaling pathway, thereby participating in tumor progression.

Our study further focused on FADS2, which had both significant differential expression and prognostic value in BC. We found that FADS2 was highly expressed in BC tissue samples and cell lines. Consistently, other research showed that FADS2 mRNA levels and activity were significantly higher in BC tissues than in normal tissues (46, 47). However, the functions of FADS2 in BC have rarely been studied and they remain unclear. In this study, functional experiments provided validation in two BC cell lines, MDA-MB-231 and BT474. We validated the role of FADS2 in the proliferation, migration, and invasion abilities of BC cells.

To conclude, we herein systematically and comprehensively evaluated the role of FADSs in BC. The mRNA and protein expression levels of various FADSs were found to be either up- or downregulated in BC, and FADS2 and FADS4 expression were closely related to tumor grade. Moreover, the genes encoding FADSs showed different degrees of genetic alterations and were significantly correlated with tumor-infiltrating immune cells. In addition, we highlighted the association between FADS2 and proliferation, migration, and invasion of BC cells. We believe that FADS1/2/3/4 are predictors of poor prognosis in patients with BC and can serve as potential therapeutic targets. Future studies should give higher importance to FADS2 and other FADS family members and further explore their potential mechanisms as well as clinical applications in BC. This study has some limitations. The protein expression levels of FADSs in the HPA database were analyzed, but we could not retrieve accurate staining statistics results for the protein expression levels of FADSs because of an insufficient number of paracarcinoma tissues in the database. Besides, immunohistochemistry data for FADS7/8 were not present in the HPA database. Accordingly, further studies need to be conducted urgently.

## Data availability statement

The original contributions presented in the study are included in the article/**Supplementary Material**. Further inquiries can be directed to the corresponding authors.

## Ethics statement

The studies involving human participants were reviewed and approved by Ethics Approval Committee of Southwest Hospital Affiliated with Third Military Medical University, Chongqing, China. The patients/participants provided their written informed consent to participate in this study.

## Author contributions

XQ, YZ and CC contributed to the conception of the study. TZ contributed significantly to the analysis and manuscript preparation. TZ, PG and YL performed the data analyses and wrote the manuscript. HT and NS helped perform the data analysis with constructive discussions. All authors contributed to the article and approved the submitted version.
