# Supplementary material for: Investigating the role of FADS family members in breast cancer based on bioinformatic analysis and experimental validation
Source: Front Immunol. 2023 Apr 12;14:1074242. doi: 10.3389/fimmu.2023.1074242 (PMC10130515; doi:10.3389/fimmu.2023.1074242)
Supplement: Supplementary file 1 [file DataSheet_1.docx]

**Supplementary Material**

| Patients | Age | Molecular Subtype | Histological Type | Stage | Surgery | ALN (Positive/ Total) | Ki-67 | Neoadjuvant Chemotherapy |
| --- | --- | --- | --- | --- | --- | --- | --- | --- |
| 1 | 63 | TNBC | NSIBC | IIA | TM+SLNB | 0/4 | 40%+ | NO |
| 2 | 56 | TNBC | NSIBC | IIIA | MRM | 9/41 | 15%+ | NO |
| 4 | 68 | TNBC | NSIBC | IIB | MRM | 3/18 | 80%+ | NO |
| 5 | 68 | TNBC | NSIBC | IIA | TM+SLNB | 0/4 | 50%+ | NO |
| 6 | 52 | Luminal A | NSIBC | IA | MRM | 0/19 | 15%+ | NO |
| 7 | 48 | HER2+ | NSIBC | IIB | MRM | 2/30 | 60%+ | YES |
| 9 | 48 | Luminal B | ILC | IIC | MRM | 10/12 | 50%+ | NO |

Table S1 Clinicopathological Characteristics of Patients.

Abbreviations: ALN, axillary lymph nodes; NSIBC, nonspecific invasive breast carcinoma; ILC, invasive lobular carcinoma; TNBC, triple negative breast cancer; TM, total mastectomy; MRM, modified radical mastectomy; SLNB, sentinel lymph node biopsy.

Table S2 Overview of FADSs.

| Gene | Description | Alias | Substrate and catalytic activity |
| --- | --- | --- | --- |
| FADS1 | Fatty acid desaturase 1 | D5D | 20:3n-6; 20:4n-3  Δ5 desaturase activity |
| FADS2 | Fatty acid desaturase 2 | D6D | 18:2n-6; 18:3n-3; 20:2n-6;  20:3n-3; 24:4n-6; 24:5n-3; 16:0  Δ6 desaturase activity  Δ8 desaturase activity |
| FADS3 | Fatty acid desaturase 3 | CYB5R, LLCDL3 | Trans 11-C18:1 (Δ13 desaturase activity) Atypical cytotoxic 1-deoxysphingosine  (1-deoxyribonucleic acid, m18:0)  (Δ 14z long chain base desaturase activity) |
| FADS4 | Stearoyl-CoA desaturase 5 | SCD5 | 16:0; 18:0  Δ9 desaturase activity |
| FADS5 | Stearoyl-CoA desaturase (delta-9-desaturase) | SCD | 16:0; 18:0  Δ9 desaturase activity |
| FADS6 | Fatty acid desaturase 6 | FP18279 | 22:5 n-3  Δ4 desaturase activity |
| FADS7 | Delta (4)-desaturase, sphingolipid 1 | DEGS1 | Sphingolipid dihydroceramide  Δ11 desaturase activity  Δ4E desaturase activity |
| FADS8 | Delta (4)-desaturase, sphingolipid 2 | DEGS2 | Phytosphingolipid  Δ4-desaturase/C4-hydroxylase |

Table S3 Summary of expression levels of FADSs in BC.

|  | FADS1 | FADS2 | FADS3 | FADS4 | FADS5 | FADS6 | FADS7 | FADS8 |
| --- | --- | --- | --- | --- | --- | --- | --- | --- |
| TCGA | Upregulated  NA | Upregulated  *** | Downregulated  *** | Downregulated  *** | Downregulated  *** | Upregulated  * | Upregulated NA | Upregulated  *** |
| GSE42568 | —— | Upregulated  * | Downregulated  *** | Upregulated  NA | Downregulated  *** | —— | Downregulated  NA | Upregulated  *** |
| GSE65194 | —— | Upregulated  ** | Downregulated  *** | Downregulated  *** | Downregulated  NA | —— | Upregulated  * | Upregulated  NA |

^NA^*P* > 0.05, **P* < 0.05, ***P* < 0.01, ****P* < 0.001

Table S4 Summary of Pearson and *P* values.

| Gene | Copy Number | | Copy Number Variants | | Deletion | | Amplification | | |
| --- | --- | --- | --- | --- | --- | --- | --- | --- | --- |
|  | cor | *P* | cor | *C* | cor | *P* | cor | | *P* |
| FADS1 | 0.23 | 1.1e-14 | 0.35 | 1.5e-13 | 0.22 | 0.0017 | 0.24 | | 0.00027 |
| FADS2 | 0.17 | 2.8e-08 | 0.26 | 8.7e-08 | 0.28 | 2.4e-05 | 0.0063 | | 0.93 |
| FADS3 | 0.26 | <2.2e-16 | 0.36 | 8.8e-15 | 0.3 | 4.5e-06 | 0.11 | | 0.13 |
| FADS4 | 0.13 | 2.9e-05 | 0.2 | 4.3e-05 | 0.042 | 0.47 | 0.17 | | 0.073 |
| FADS5 | 0.039 | 0.21 | 0.12 | 0.015 | 0.12 | 0.034 | -0.018 | | 0.87 |
| FADS6 | 0.12 | 5.9e-05 | 0.1 | 0.014 | 0.08 | 0.3 | | 0.11 | 0.034 |
| FADS7 | 0.39 | <2.2e-16 | 0.33 | <2.2e-16 | 0.27 | 0.19 | | 0.26 | 5e-14 |
| FADS8 | 0.29 | <2.2e-16 | 0.33 | <2.2e-16 | -0.012 | 0.83 | | 0.078 | 0.34 |

cor, correlation

Table S5 Summary of the correlation between the expression level of FADSs and FADS-targeting miRNAs, as well as the expression of miRNAs in BC.

| Gene | miRNA name | cor | *P* | Fold Change | *P-*Value |
| --- | --- | --- | --- | --- | --- |
| FADS1 | hsa-miR-130b-3p  hsa-miR-197-3p  hsa-miR-345-5p  hsa-miR-324-5p  hsa-miR-301a-3p  hsa-miR-222-3p  hsa-miR-532-3p  hsa-miR-1224-5p  hsa-miR-186-5p  hsa-miR-454-3p  hsa-miR-18a-5p  hsa-miR-105-5p  hsa-miR-1271-5p  hsa-miR-140-5p  hsa-let-7i-5p  hsa-miR-182-5p | 0.35  0.289  0.282  0.265  0.261  0.255  0.248  0.239  0.22  0.211  0.208  0.207  0.206  0.203  0.201  -0.203 | 1.29E-32  2.55E-22  3.06E-21  7.00E-19  2.39E-18  1.40E-17  1.15E-16  1.44E-15  2.39E-13  2.03E-12  4.31E-12  5.39E-12  6.52E-12  1.36E-11  2.47E-11  1.50e-11 | 3.29  0.89  2.43  1.43  3.92  1.03  0.97  4.92  0.95  2.8  3.01  73.71  0.57  0.69  0.86  4.94 | 1.40E-38  0.00077  6.70E-10  0.00058  4.90E-24  0.0015  0.006  0.00018  0.0062  6.20E-30  9.30E-09  1.50E-09  3.20E-11  3.70E-14  9.10E-07  2.10E-86 |
| FADS2 | hsa-miR-130b-3p  hsa-miR-324-5p  hsa-miR-301a-3p  hsa-miR-1301-3p  hsa-miR-9-5p  hsa-miR-532-3p  hsa-miR-105-5p  hsa-miR-148b-3p  hsa-miR-874-3p | 0.333  0.268  0.267  0.261  0.229  0.22  0.213  0.212  0.212 | 1.79E-29  2.70E-19  3.33E-19  2.05E-18  2.51E-14  2.18E-13  1.26E-12  1.77E-12  1.71E-12 | 3.29  1.43  3.92  4.32  4.19  0.97  73.71  2.41  0.82 | 1.40E-38  0.00058  4.90E-24  2.30E-47  0.019  0.006  1.50E-09  2.70E-34  1.40E-07 |
| FADS3 | hsa-let-7i-5p  hsa-let-7b-5p  hsa-miR-628-5p | 0.23  -0.203  -0.226 | 1.52E-14  1.38E-11  4.91E-14 | 0.86  0.78  1.66 | 1.80E-08  0.0085  9.10E-07 |
| FADS4 | hsa-miR-379-5p | 0.23 | 1.68E-14 | 0.43 | 9.3E-33 |
| FADS5 | hsa-miR-425-5p  hsa-miR-628-5p  hsa-miR-331-3p  hsa-miR-328-3p  hsa-miR-345-5p  hsa-miR-181d-5p  hsa-miR-383-5p  hsa-miR-181c-5p | 0.299  0.236  0.208  0.202  0.201  -0.216  -0.222  -0.225 | 6.66E-24  3.62E-15  4.65E-12  1.87E-11  2.58E-11  6.99E-13  1.33E-13  5.76E-14 | 2.01  1.66  2.08  0.55  2.43  1.98  0.39  1.51 | 1.50E-13  0.0085  1.70E-11  2.40E-19  6.70E-10  2.50E-07  3.40E-43  0.00027 |
| FADS6 | NA | NA |  | NA | NA |
| FADS7 | hsa-miR-4677-3p | 0.224 | 9.25E-14 | 2.22 | 5.80E-24 |
| FADS8 | hsa-miR-148a-3p  hsa-miR-1301-3p  hsa-miR-19a-3p  hsa-miR-455-3p | -0.22  -0.361  -0.427  -0.475 | 2.53E-13  9.43E-35  2.64E-49  3.21E-62 | 1.9  4.32  2.53  2.39 | 2.70E-11  2.30E-47  1.30E-10  5.90E-07 |

Table S6 Transcription factors of FADSs.

| Key Transcription Factor | Description | Regulated Gene | *P* | *FDR* |
| --- | --- | --- | --- | --- |
| SREBF1 | Sterol regulatory element  binding transcription factor 1 | FADS4, FADS5 | <0.001 | <0.001 |

Table S7 Kinases of FADSs.

| FADSs | Kinase | Leading Edge Num | NES | *FDR* |
| --- | --- | --- | --- | --- |
| FADS1 | Kinase_CDK1 | 79 | 2.3899 | <0.001 |
|  | Kinase_CDK2 | 89 | 1.9201 | <0.001 |
|  | Kinase_CSNK2A1 | 72 | 1.8662 | <0.001 |
| FADS2 | Kinase_CDK1 | 83 | 2.4440 | <0.001 |
|  | Kinase_CDK2 | 97 | 2.0868 | <0.001 |
|  | Kinase_CSNK2A1 | 78 | 1.9223 | <0.001 |
| FADS3 | Kinase_PRKCA | 53 | 1.5354 | <0.001 |
|  | Kinase_PRKCD | 19 | 1.4104 | 0.020 |
|  | Kinase_ATM | 43 | -1.6842 | <0.001 |
|  | Kinase_CDK1 | 82 | -1.5806 | <0.001 |
|  | Kinase_CDK2 | 74 | -1.2713 | 0.019 |
| FADS4 | Kinase_PRKCA | 69 | 1.4265 | <0.001 |
| FADS5 | Kinase_CDK1 | 110 | 2.2312 | <0.001 |
|  | Kinase_CSNK2A1 | 82 | 1.6241 | <0.001 |
| FADS6 | Kinase_CDK2 | 77 | 1.5792 | <0.001 |
|  | Kinase_CDK1 | 87 | 1.4902 | <0.001 |
| FADS7 | Kinase_CSNK2A1 | 67 | -1.4751 | <0.001 |
|  | Kinase_CDK2 | 94 | -1.7593 | <0.001 |
|  | Kinase_CDK1 | 89 | -1.8180 | <0.001 |
|  | Kinase_CHEK1 | 44 | -1.8286 | <0.001 |
|  | Kinase_ATM | 46 | -1.8770 | <0.001 |
| FADS8 | Kinase_CSNK2A1 | 67 | -1.4641 | <0.001 |
|  | Kinase_CDK2 | 94 | -1.7681 | <0.001 |
|  | Kinase_CHEK1 | 44 | -1.8368 | <0.001 |
|  | Kinase_ATM | 46 | -1.8815 | <0.001 |

Table S8 Correlation analysis between FADSs and tumor-infiltrating immune cells.

|  | Purity | | B Cells | | CD8+ T Cells | | CD4+ T Cells | | Macrophages | | Neutrophils | | Dendritic Cells | |
| --- | --- | --- | --- | --- | --- | --- | --- | --- | --- | --- | --- | --- | --- | --- |
|  | cor | *P* | cor | *P* | cor | *P* | cor | *P* | cor | *P* | cor | *P* | cor | *P* |
| FADS1 | -0.003 | 0.924 | 0.166 | 1.76e-07 | 0.167 | 1.53e-07 | 0.063 | 0.101 | 0.196 | 5.94e-10 | 0.226 | 1.85e-12 | 0.216 | 1.52e-11 |
| FADS2 | 0.022 | 0.495 | 0.174 | 4.41e-08 | 0.04 | 0.212 | -0.008 | 0.793 | 0.041 | 0.201 | 0.09 | 5.31e-03 | 0.106 | 1.03e-03 |
| FADS3 | -0.232 | 1.42e-13 | -0.01 | 0.743 | -0.049 | 0.123 | 0.2 | 3.98e-10 | -0.025 | 0.436 | 0.152 | 2.53e-06 | 0.169 | 1.58e-07 |
| FADS4 | -0.157 | 6.88e-07 | 0.024 | 0.446 | 0.261 | 1.32e-16 | 0.144 | 7.47e-06 | 0.119 | 1.75e-04 | 0.179 | 2.71e-08 | 0.154 | 1.82e-06 |
| FADS5 | 0.111 | 4.76e-04 | 0.039 | 0.219 | 0.115 | 3.24e-04 | -0.137 | 1.95e-05 | 0.115 | 3.11e-04 | 0.028 | 0.394 | 0.031 | 0.333 |
| FADS7 | -0.086 | 6.79e-03 | 0.076 | 1.80e-02 | 0.16 | 4.71e-07 | 0.103 | 1.36e-03 | 0.149 | 2.51e-06 | 0.184 | 1.07e-08 | 0.147 | 4.98e-06 |
| FADS8 | 0.103 | 1.13e-03 | -0.147 | 4.00e-06 | -0.125 | 9.06e-05 | -0.078 | 1.54e-02 | 0.053 | 0.0944 | -0.146 | 6.55e-06 | -0.184 | 1.17e-08 |


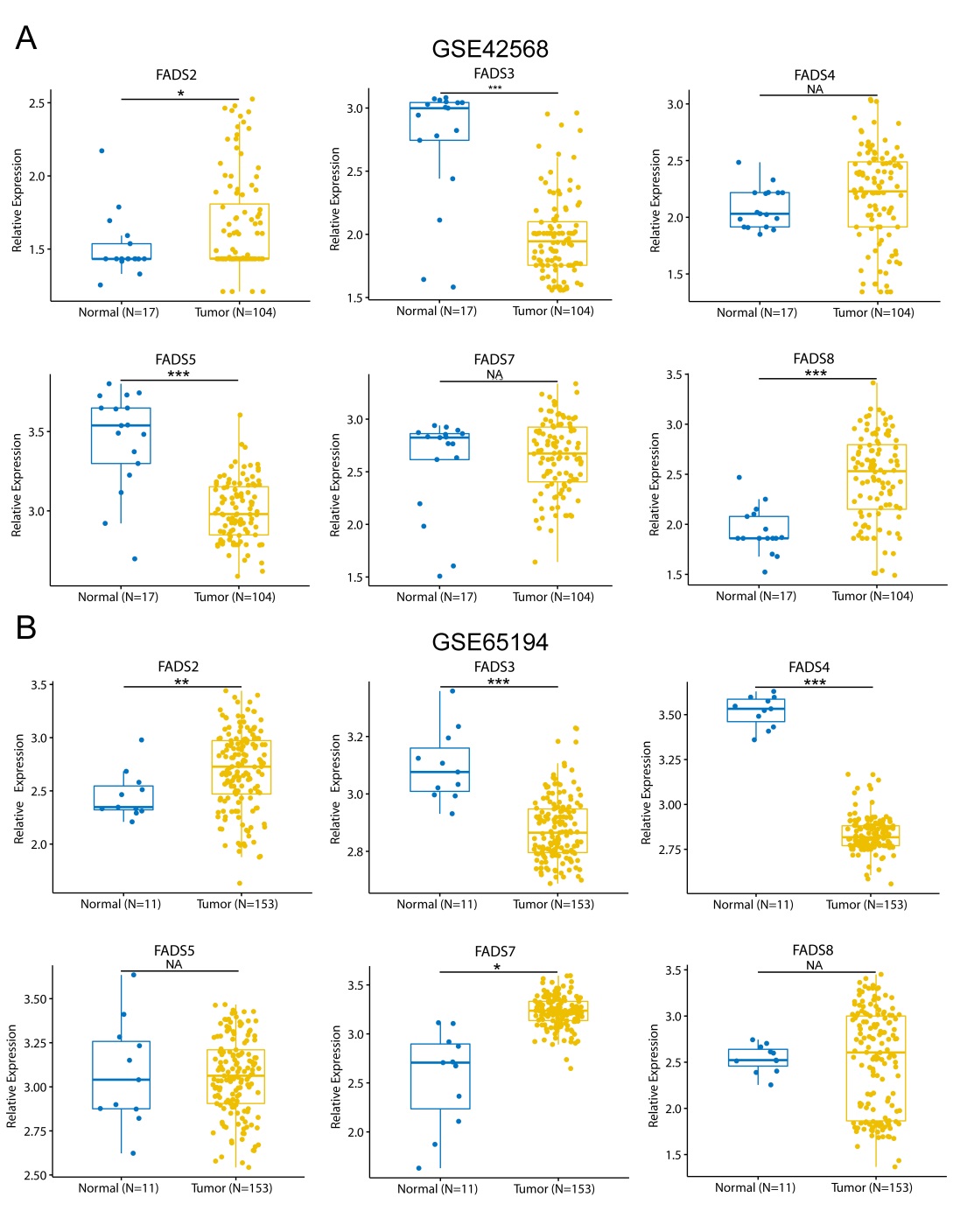


Figure S1 mRNA expression levels of FADSs in BC were validated in GSE65194 (A) and GSE42568 (B) (^NA^*P* > 0.05, ^*^*P* < 0.05, ^**^*P* < 0.01, ^***^*P* < 0.001).


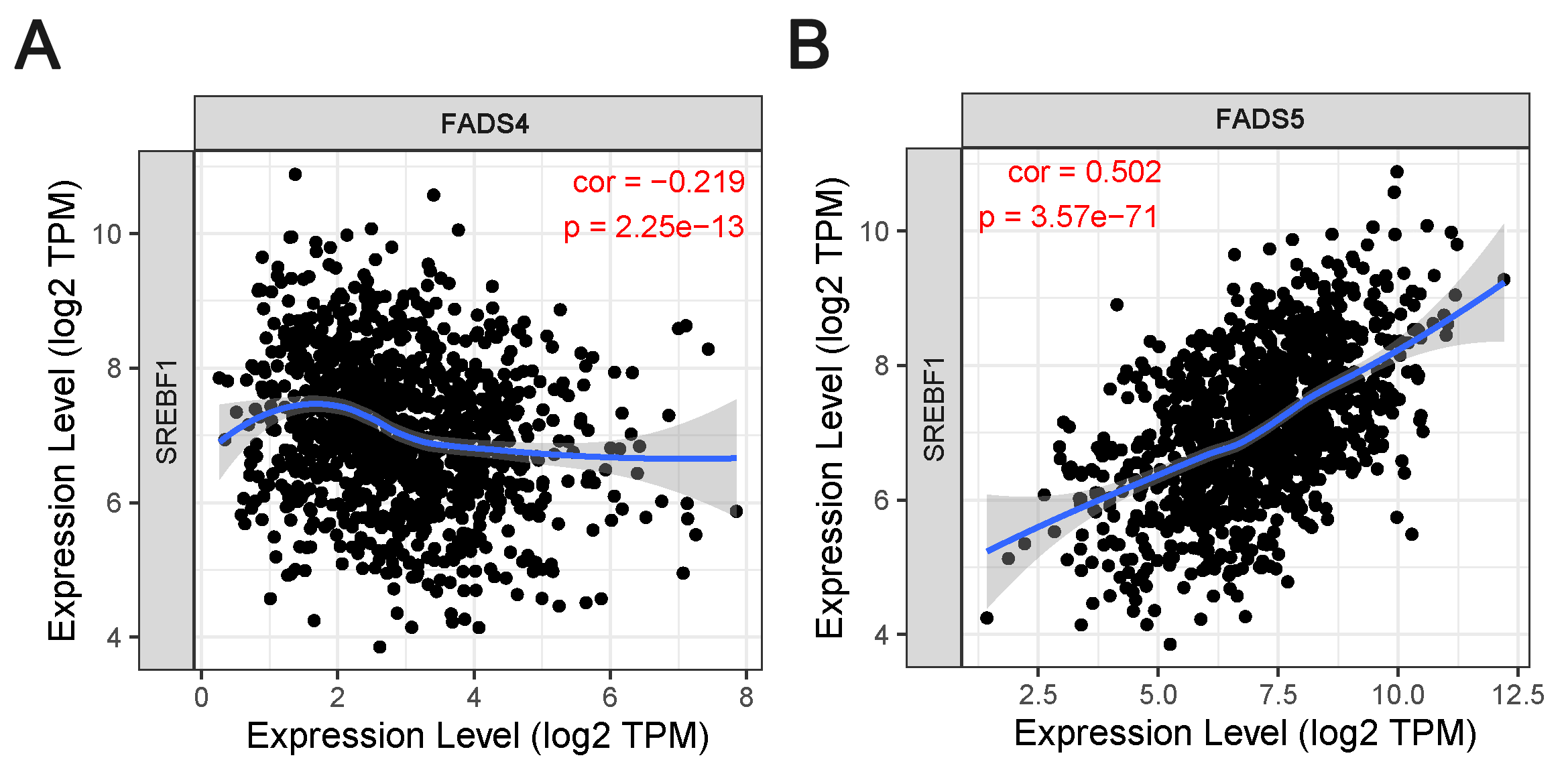


Figure S2 The correlation between FADS4/5 and SREBF1 in BC. cor, correlation.


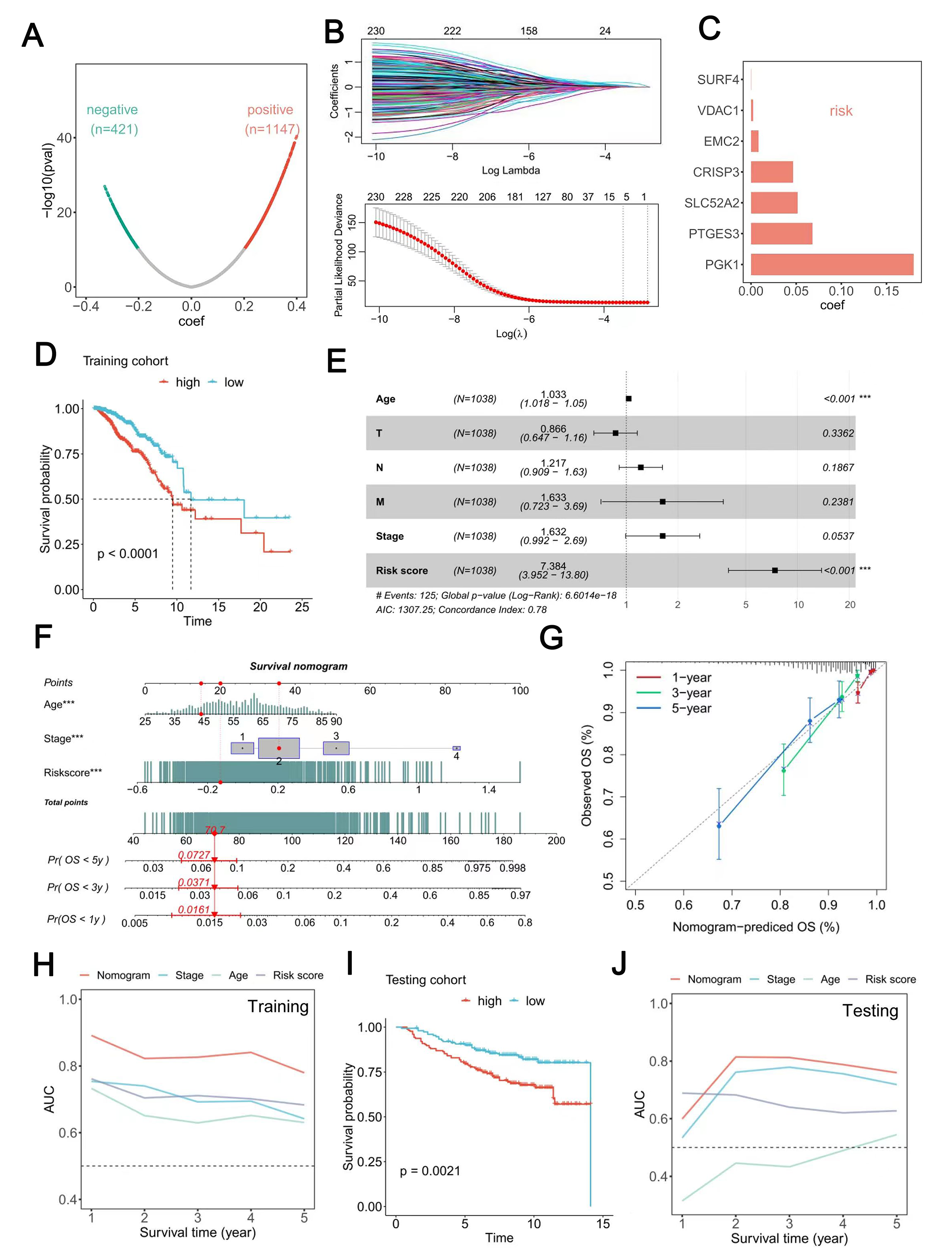


Figure S3 Construction and validation of prognostic risk model. (A)Scatter plot of FADS2-associated genes in the training cohort with the selection threshold as |Pearson coefficient| > 0.2 and p-value < 0.001. (B) Lasso Cox regression analysis was performed and 7 genes were gathered to establish the prognostic model. (C) Multivariate Cox regression analysis was carried out sequentially and 7 associated genes significantly correlated with the prognosis of patients. Riskscore=(0.0466)*CRISP3+(0.0083)*EMC2+(0.1801)*PGK1+(0.0681)*PTGES3+(0.0516)*SLC52A2+(0.0004)*SURF4+(0.0024)*VDAC1. (D) Survival plot between high-risk group and low-risk group (median risk score as cut-off value). (E) Multivariate cox regression in training cohort. (F) Nomogram was constructed to predict the 1-year, 3-year and 5-year survival based on age, pathological stage, and risk score in the TCGA training cohort. (G) Calibration curves to show the consistency between ideal survival prediction and actual survival prediction based on the nomogram. (H) Comparison of the time-dependent AUC between nomogram and various clinical parameters. (I) Survival plot in testing cohort GSE20685 (n = 327) and the same cut-off value of risk score was employed. (J) Comparison of the time-dependent AUC between nomogram and various clinical factors in the testing cohort GSE20685 (n = 327). ^***^*P* < 0.001
